# Cyberchondria: An emerging entity in COVID-19 pandemic and thereafter

**DOI:** 10.1192/j.eurpsy.2021.799

**Published:** 2021-08-13

**Authors:** U. Bhaumik, S. Nayok

**Affiliations:** 1 Psychiatry, Independent practice, Kolkata, India; 2 Psychiatry, Sri Siddhartha Medical College, Tumkur, India

**Keywords:** COVID-19, pandemic, cyberchondria, health anxiety

## Abstract

**Introduction:**

Cyberchondria is a pathological behaviour linked to excessive online searching of health information.It is frequently associated with health anxiety. It may be regarded as an compulsive behaviour secondary to obsessions about a real or an imagined illness. The coronavirus pandemic of 2019 has brought about a fear of getting infected. In the absence of a definitive cure, the focus largely lies on stringent preventive measures and early diagnosis. Known to present with diverse symptoms, fear of coronaviral infection makes affected individuals search for symptoms on internet for reassurance. Added misinformation further increases stress,anxiety and confusion.

**Objectives:**

The authors attempt to describe cyberchondria and highlight its increased prevalence during the coronavirus pandemic.

**Methods:**

5 cases from different backgrounds were seen in the outpatient clinic during the months of April-July 2020.Consent was obtained from subjects before the study.They were clinically diagnosed with obsessive-compulsive disorder and exhibited cyberchondria in the background of the pandemic.

**Results:**

All of the described 5 cases had prominent fear of contracting or having contracted coronavirus disease-19.All of them were found to have significant scores rang on Yale-Brown Obsessive-Compulsive Severity Scale(ranging from 25-35) and improved after a trial of selective serotonin reuptake inhibitors.

**Conclusions:**

The emergence of cyberchondria during the coronavirus -19 pandemic warrants further introspection.Changes in policy-making to prevent misinformation and present health information in a simple way to prevent confusion in the lay public is a necessity to tackle this problem in the near future.

